# Gastric dilatation and intestinal obstruction mimicking acute coronary syndrome with dynamic electrocardiographic changes

**DOI:** 10.1186/s12872-016-0423-z

**Published:** 2016-11-29

**Authors:** H. M. M. T. B. Herath, Anne thushara Matthias, B. S. D. P. Keragala, W. A. E. Udeshika, Aruna Kulatunga

**Affiliations:** National Hospital, Colombo, Sri Lanka

**Keywords:** Acute coronary syndrome, Dynamic electrocardiographic changes, Gastric dilatation, Intestinal obstruction

## Abstract

**Background:**

ST elevation myocardial infarction is a medical emergency and the electrocardiogram is a part of the mainstay in the initial diagnosis. A variety of non-cardiac conditions have been known to mimic the electrocardiographic changes seen in acute coronary syndrome. We present a patient presenting with acute partial intestinal obstruction causing gastric distension and intestinal dilatation who also had dynamic electrocardiographic changes, mimicking anterior ST elevation myocardial infarction. Only very few cases of gastric distention and intestinal dilatation leading to acute ST segment elevation in electrocardiogram are reported so far in literature.

**Case presentation:**

A fifty-six-year-old Sri Lankan male, without any modifiable risk factors for ischemic heart disease presented with acute onset nausea, vomiting, sweating, abdominal discomfort and fullness without any chest pain. On examination, he had a pulse rate of 50 beats per minute and his blood pressure was 110/50 mmHg. His abdomen was distended and the liver dullness was not detectable. Subsequent ECG showed > 2 mm ST elevations with T inversions in chest leads V1 to V3, J point elevation in leads L *11*, L *111*, aVF and T inversion in leads L *1 and aVL*. Cardiac biomarkers were normal and 2D echo showed normal left ventricular function without any regional wall motion abnormalities. Abdominal X-ray showed a distended stomach, dilated ascending and descending colon with absent rectal air. Electrocardiographic changes reverted back to normal with the resolution of bowel obstruction.

**Conclusion:**

The mechanism of ECG changes in such a case like this is yet to be elucidated, but can be postulated to happen due to change in the position of the heart in the thoracic cavity causing change in the cardiac axis. This case emphasizes the importance of a proper history and highlights the value of auxiliary investigations such as cardiac biomarkers and echocardiogram in the diagnosis of acute coronary syndrome in a confusing situation such as this. This also illustrates the importance of early recognition of other noncardiac causes like acute gastric distention as being responsible for dynamic ECG changes. This will obviate a myriad of unnecessary investigations, interventions, costly management strategies and patient anxiety.

## Background

Myocardial infarction (MI) is defined as a clinical or pathological event due to myocardial ischemia causing myocardial injury or necrosis and the electrocardiogram (ECG) is a mainstay in the initial diagnosis of patients with suspected acute coronary syndrome (ACS). ST elevation Myocardial infarction (STEMI) is a medical emergency, since the beneficial effects of therapy with reperfusion are greatest when performed early. But a variety of noncardiac conditions have been known to mimic the ECG changes seen ACS including cholecystitis [1–4], pancreatitis [5, 6], pneumonia [7], gastric distention [8, 9], acute stroke, subarachnoid hemorrhage [10], pericarditis, neoplastic invasion of the myocardium, acute pulmonary embolism, and hypothermia. Here we present a male patient presenting with acute partial intestinal obstruction causing gastric distension and intestinal dilatation with dynamic ECG changes mimicking anterior STEMI. This can be explained by a change in the heart's position secondary to gastric distention causing a shift in the mean QRS axis, stress-related/catecholamine-associated cardiomyopathy, and by an irritative/direct compression effect on the heart from gastric distention. ECG changes resolved with resolution of intestinal obstruction. Prompt recognition of these noncardiac causes may reduce morbidity, mortality, unnecessary interventions, treatment expenditure and patient anxiety.

## Case presentation

A fifty-six-year-old Sri Lankan male patient presented with acute onset nausea, vomiting and sweating associated with abdominal discomfort and fullness. He did not complain of colicky abdominal pain. Even though he was passing flatus, he had not opened his bowels for 2 days. He did not have any chest pain, palpitations or shortness of breath. There was no loss of consciousness or any other focal neurological symptoms. He had three similar episodes in the past, latest of which was 20 years prior. All of them had resolved spontaneously and no records were available. Barring that, his past medical details and family history were unremarkable. He did not have any history of altered bowel habits, per rectal bleeding or family history of colorectal carcinoma. He was a nonsmoker and a teetotaler.

On examination he was pale with a pulse rate of 50 beats per minute and his blood pressure was 110/50 mmHg. Abdomen was distended and the liver dullness was absent. There was no tenderness, guarding or rigidity. Bowel sounds were reduced and sluggish. There were no focal neurological signs.

**Fig. 1:**
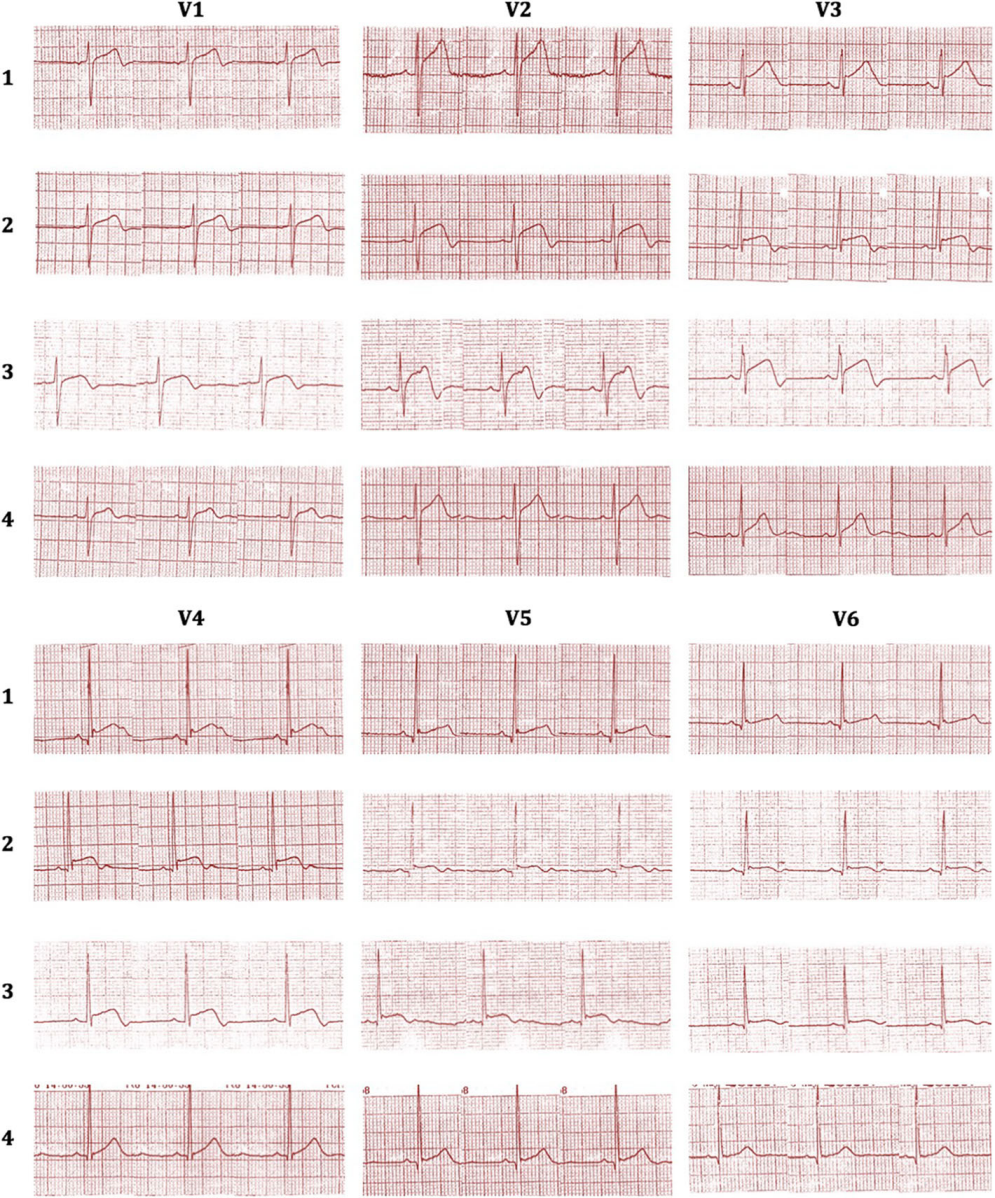
Dynamic ST segment elevations with T inversion in chest leads V1 to V3 (legend = 1 - On admission (three hours after the beginning of symptoms), 2 - Fifteen minutes after the admission, 3 - Six hours after the admission, 4 - Twelve hours after the admission)

**Fig. 2:**
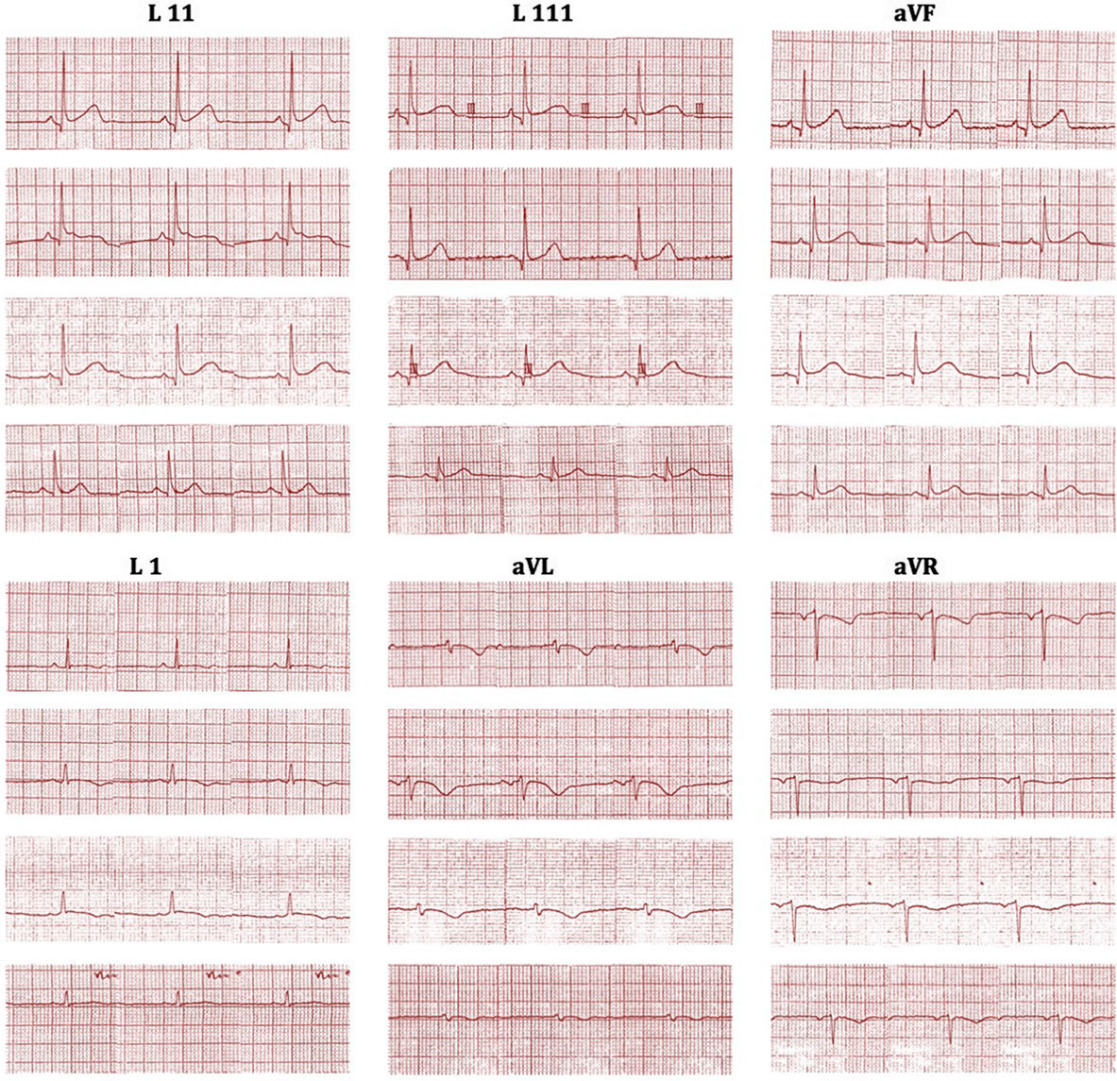
J point elevation in leads L *11*, L *111*, and aVF and dynamic T inversion in leads L *1 and aVL* (legend = 1 - On admission (three hours after the beginning of symptoms), 2 - Fifteen minutes after the admission, 3 - Six hours after the admission, 4 - Twelve hours after the admission)

**Fig. 3:**
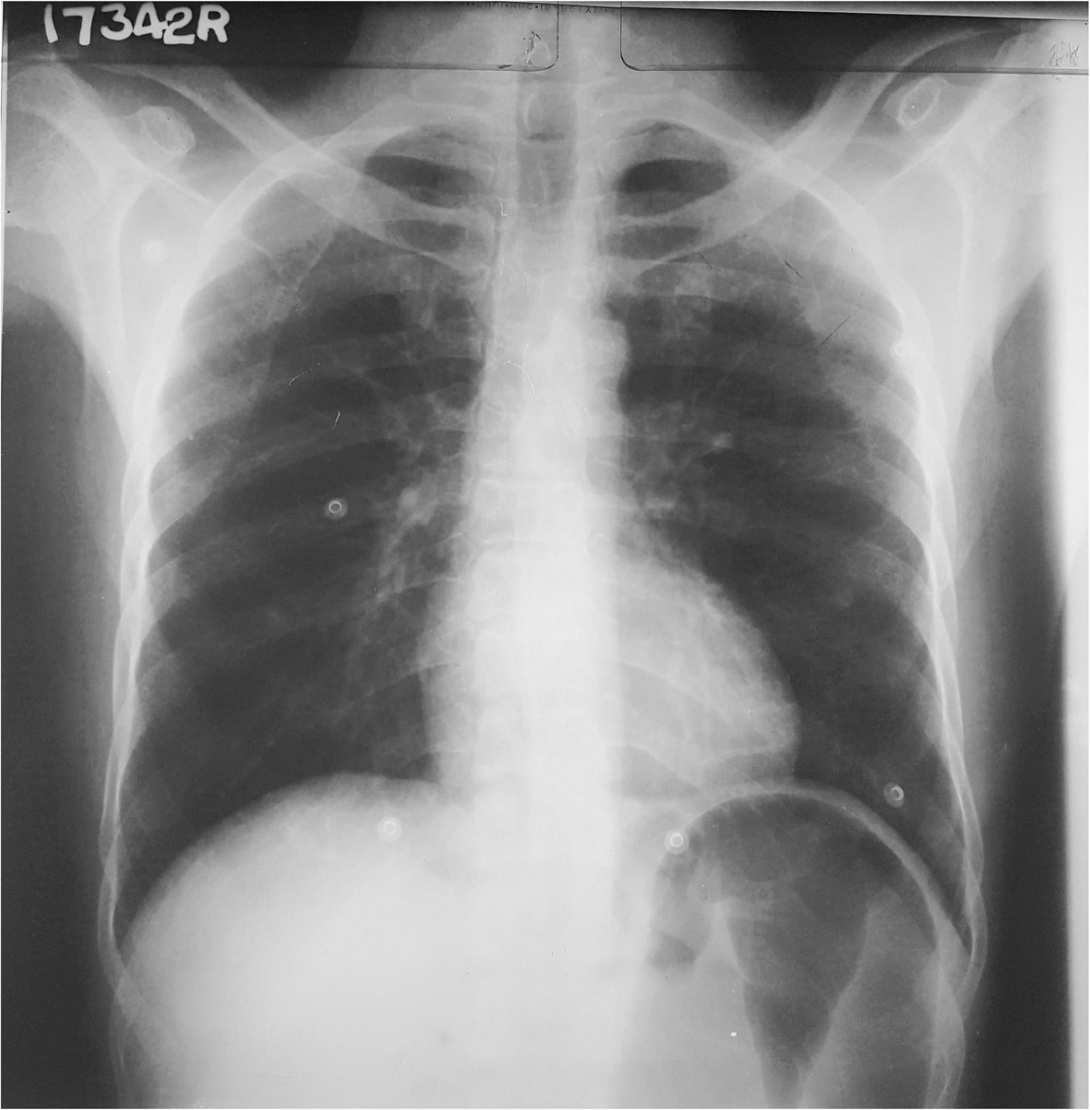
CXR – PA showing distended stomach

**Fig. 4:**
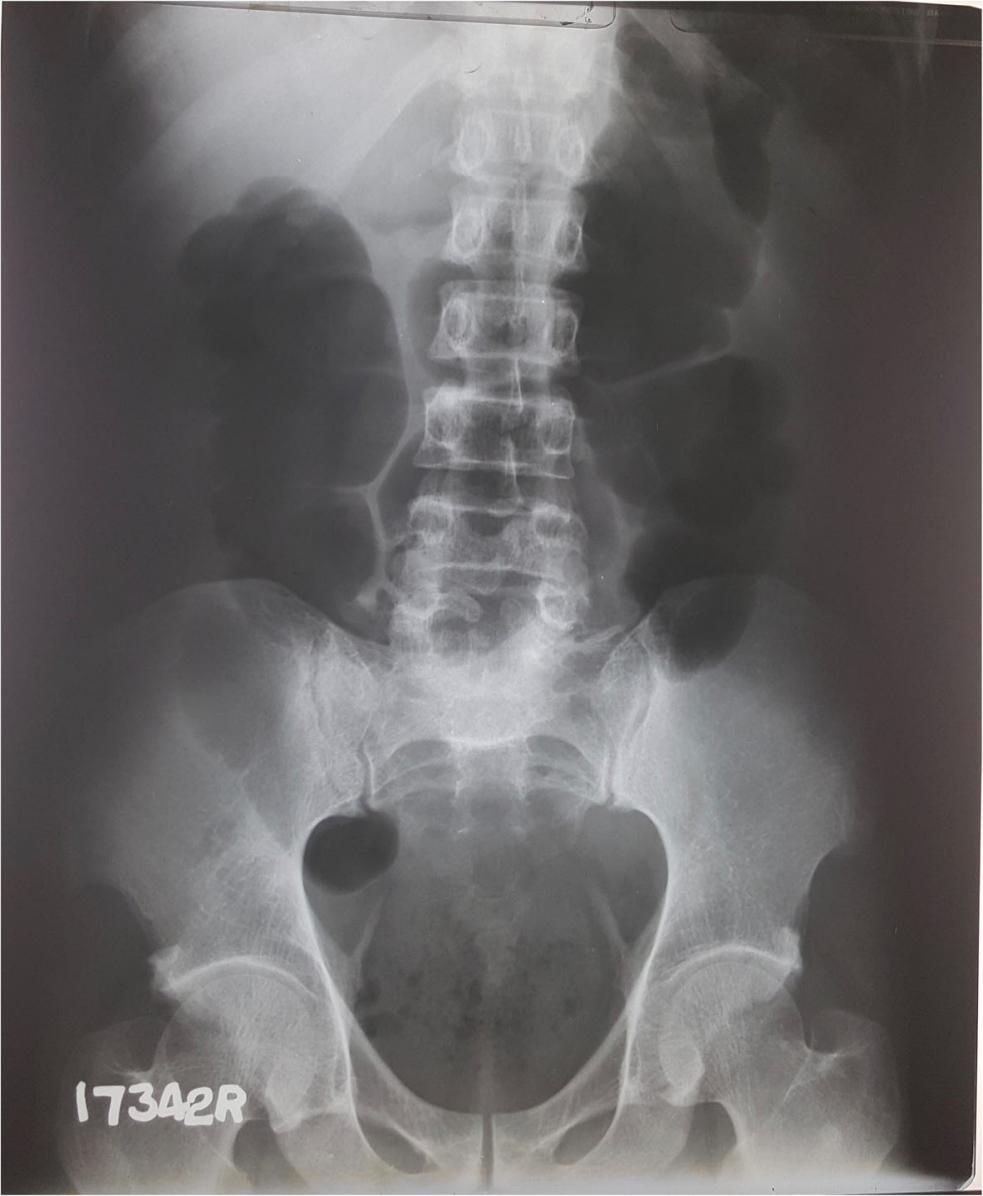
X-ray abdomen supine showing dilated ascending and descending colon with absent rectal air

ECG done soon after the admission (3 h after the onset of symptoms) showed dynamic ST segment elevation with T inversion in leads V1 to V3, J point elevation in leads L *11*, L *111*, aVF and T inversion in leads L *1 and aVL* (Figs. 1 and 2). The changes were progressive in serial ECGs. 2D Echocardiogram revealed normal left ventricular function with normal ejection fraction without any regional wall motion abnormalities or pericardial effusion. Three troponin I titers done at 4, 12 and 24 h from the onset of symptoms were < 0.10 ng/ml (less than 0.5 ng/ml) with CK-MB = 3.34 ng/ml, 3.88 ng/ml, 3.45 ng/ml (<5) and the myoglobin levels were = 29.94 ng/ml, 31.45 ng/ml, 18.65 ng/ml (<80) respectively. Abdominal X-ray in erect position and Chest x-ray PA done 6 h after the onset of symptoms showed distended stomach, dilated ascending and descending colon with absent rectal air (Figs. 3 and 4). There was no volvulus or air under the diaphragm. Ultrasound scan of the abdomen showed no hepatomegaly, splenomegaly, abdominal masses or free fluid. There was no ultrasonographic evidence of acute cholecystitis or acute pancreatitis. NCCT brain was normal. Full blood count, serum electrolytes, renal function tests and liver function tests were normal (Table 1). Serum amylase was 179 U/L (22–80) and CRP was 10 mg/dL (<6) TSH was 1.4 mIU/L (0.55–4.78) and Free T4 1.12 ng/dL (0.89–1.76) Fasting blood sugar was 5.5 mmol/L and Lipid profile was normal.

**Table 1:** Full blood count, serum electrolyte and renal function tests and liver function tests

WBC 10.5 × 10^3^/μL	Neutrophil 88.4%	Lymphocyte 7.5%	Eosinophil 0.02%
Heamoglobin 13.9 g/dL	RBC 4.3 10^6^/μL	HCT 42.7%	Platelet 187 10^3^ ×/μL
Serum creatinine 1.26 mg/dL	Serum sodium 137 mmol/L	Serum potassium 3.7 mmol/	Serum chloride 99 mmol/L
Albumin 40 g/L	Globulin 26 g/L	Alkaline phosphatase 72 U/L	Total bilirubin 14.2 μmol
Ionized calcium 1.2 mmol/L (1.0–1.3)		Serum magnesium 1.1 mmol/L (0.8–1.1)	

The patient was managed as acute partial intestinal obstruction with monitoring and supporting care. Naso gastric tube was inserted and the patient was kept nil by mouth. Vomiting and abdominal pain settled in 12 h and vital parameters normalized. Oral feeding was gradually introduced. ECG changes reverted back to normal without any residual ischemic changes within this period. Repeat abdominal x-ray was also normal. He was discharged on the fifth day, with colonoscopy planned for a later date.

## Discussion

ACS is due to ischemia of the cardiac muscles and is a medical emergency. Prompt recognition of the condition with a proper history together with an ECG and cardiac biomarkers is of utmost importance. Despite a variety of available diagnostic tests, ECG remains the mainstay of initial diagnosis and vascular territory determination in patients with suspected ACS. Several noncardaic conditions can cause ECG changes that can mimic ACS due to positional changes of the heart within the thoracic cavity, temperature effects on the heart, abnormal neurologic input to the heart, hormonal abnormalities, increased pressure within the cardiovascular system and interposition of fluid or tissues between the heart and the ECG electrodes [11].

Here we describe a patient who did not have any modifiable risk factors for ischemic heart disease presenting with features of acute partial intestinal obstruction and dynamic ECG changes. His x-rays showed gaseous distention of the stomach with dilated bowel loops. ECG taken on admission, at 15 min and 4 h from the admission, showed dynamic ST segment elevation with T inversion in leads V1 to V3, T inversion in leads L1, aVL and J point elevations in inferior leads. These changes resolved to normal within 12 h. He did not have any chest pain and the cardiac biomarkers and the 2D echo were normal which excluded ST elevation MI.

Other than myocardial ischemia, ECG changes can be caused by other cardiac (myocarditis, pericarditis, early repolarization, ventricular hypertrophy, bundle branch blocks, cardiomyopathies, neoplastic invasion of the myocardium and ventricular aneurysms) and noncardaic causes. Acute cholecystitis has been reported to cause ECG changes and alteration of the coronary blood supply due to gall bladder distension might be the causal mechanism for this association [1–4]. Nonspecific T-wave changes and accelerated atrial or nodal rhythms have been reported in acute pancreatitis. Unmasking of underlying ischemic heart disease by the stress of acute pancreatitis and the imbalance of autonomic nervous system had been considered as possible mechanisms for those ECG changes [5, 6]. Acute and reversible electrocardiographic changes are common in acute community-acquired lobar pneumonia [7]. ECG abnormalities frequently occur with aneurysmal subarachnoid hemorrhage and these morphological waveform changes and arrhythmias are often unrecognized or misinterpreted, potentially placing patients at risk for inappropriate management [10]. Acute stroke, traumatic brain injury, esophageal perforation, splenic rupture, hiatal hernia, acute pulmonary embolism, acute aortic dissection, lung cancer, spontaneous pneumothorax and hypothermia are also known to cause ECG changes.

Dramatic electrocardiographic T-wave changes with gastric dilatation without any evidence of coronary artery disease have been reported [8]. One case report of an adolescent male following trauma illustrated the need to consider acute gastric distention in the differential diagnosis of acute ST segment elevations in the ECG [9]. In this instance, the ST segment elevations had become isoelectric following gastric decompression. Another case report described a 64-year-old woman presenting with abdominal pain and intestinal Obstruction and the electrocardiogram was suggestive of a possible acute antero-septal myocardial infarction. However these changes had resolved following nasogastric suction along with her symptoms [12]. Inferolateral ST-segment elevation on a 12-lead ECG in a 42-year-old female, which had resolved rapidly after surgical decompression of intestinal distension, was also found in the literature [13].

Several possible mechanisms can explain these ECG changes which occur in intestinal obstruction and gastric dilatation.

1. Change in the position of the heart in the thoracic cavity secondary to gastric distention.
2. Elevated vagal tone due to pain causing coronary artery spasms.
3. Stress-related catecholamine-associated cardiomyopathy.
4. Variant angina.
5. An irritative effect on the heart by the distended stomach.
6. Compression of the anterior surface of the heart (right atrium and ventricle) and possibly the right ventricular marginal branches by the distended stomach.

ECG changes secondary to changes of position of the heart and diaphragm have been described in literature. This can be due to a shift in the mean QRS axis resulting from displacement of the heart causing changes in voltage or to a change in the type or quantity of tissue between the chest wall and the heart. Duke, in 1965, demonstrated a leftward shift of the QRS axis in 12 healthy volunteers following gastric distention using rapid instillation of air, which lead to a shift in the cardiac position in the chest [14]. In restrictive and obstructive lung diseases, changes in the position of the diaphragm affects the right atrium because the right atrium is necessarily carried by attachments to the right diaphragmatic leaf. This contributes to or causes different P-axis orientations [15]. An unusual case of electrical alternans due to diaphragmatic eventration has also being described [16].

Initially the heart rate was low in our patient possibly due to increased vagal tone. Visceral-cardiac reflex secondary to gastric distention which causes increased vagal tone can lead to ECG changes. Symmetrical T-wave inversions in patients with biliary pathology have been explained using this concept in several case reports [3, 4]. The increased vagal tone may also cause transient coronary vasospasm.

Another rare differential diagnosis is stress-related cardiomyopathy, including Takotsubo syndrome. In Takotsubo syndrome it is more common to see ECG changes in the precordial leads. How ever in our patient, the ECG changes resolved within several hours as opposed to a few days or longer with Takotsubo syndrome. He did not have features of heart failure and there was no echocardiographic evidence suggestive of Takotsubo syndrome.

Variant angina, which is also referred to as Prinzmetal or coronary vasospastic angina is a clinical entity which is associated with ST-segment elevation. The precipitation of spasm by acetylcholine and methacholine suggests a role for an imbalance of vagal and sympathetic tone which can trigger coronary spasms [17]. Our patient did not have any chest pain and there was no risk factors for Prinzmetal angina. Kounis–Zavras syndrome/allergic angina or myocardial infarction is a very rare clinical entity where an allergic insult results in coronary vasospasm. Here the exact pathophysiology is not known and inflammatory mediators released in the setting of anaphylactic reactions appear to be the primary mechanism leading to allergic myocardial infarction [18]. Coronary artery anomalies can also present as atypical chest-pain syndrome and presenting with STEMI is really uncommon. The integration between traditional coronary artery angiography and multidetector computerized tomography is crucial to optimize the interventional and medical management of these patients [19].

Of the multitude of possibilities described above, it is unlikely that our patient had Brugada syndrome, stress-related cardiomyopathy or prinzmetal angina. Therefore most probably the ECG changes were due to a change in the position of the heart in the thoracic cavity causing changes in the cardiac axis and due to increased vagal tone causing transient coronary artery spasm.

Diagnosing ACS depends on obtaining an accurate medical history, cardiac enzyme test results and interpretation of the ECG. This case also emphasizes the importance of the history in diagnosing ACS as our patient did not have any chest pain suggesting MI. Cardiac biomarkers are also important in the diagnosis of ACS.

## Conclusion

Here we present a middle age male patient with intestinal obstruction causing gastric distension and intestinal dilatation who was detected to have dynamic ECG changes mimicking acute anterior STEMI in the setting of normal cardiac biomarkers and 2D Echo. This is most probably due to change in the position of the heart in the thoracic cavity causing change in the cardiac axis and increased vagal to leading to coronary artery spasms. This case also emphasizes the importance of the proper history in diagnosing ACS as our patient did not have any chest pain suggesting MI. Cardiac biomarkers and 2D echo are also important in the diagnosis of ACS. This case illustrates the need to consider and recognize early acute gastric distention and other noncardiac causes for dynamic ECG changes to avoid a series of unnecessary investigations and costly management strategies.

## Abbreviations

ACS: Acute coronary syndrome
ECG: Electrocardiogram
MI: Myocardial infraction
NCCT: Non contrast computed tomography
STEMI: ST elevation myocardial infarction
